# The classification of handwriting features of the Kazakh language written in Latin script

**DOI:** 10.1080/20961790.2021.1963203

**Published:** 2022-04-13

**Authors:** Akyldana Galymzhanova, James Gooch, Nunzianda Frascione

**Affiliations:** a Forensic Examinations Center of the Ministry of Justice of the Republic of Kazakhstan, Nur-Sultan, the Republic of Kazakhstan; b Department of Analytical, Environmental and Forensic Sciences, King’s College London, London, UK

**Keywords:** Forensic sciences, handwriting examination, Kazakh, Latin, frequency occurrence, discriminating power, macro features, micro features

## Abstract

In 2017, the Republic of Kazakhstan began the phased transition of its alphabet from Cyrillic to Latin script. This transition has presented significant challenges to Kazakhstani document examiners, who have yet to develop appropriate methodologies for the analysis of handwriting samples written in the Kazakh language using Latin letters. This study aims to identify distinguishing macro and micro features of letters within Kazakh writing samples produced using the Latin alphabet and determine their frequencies of occurrence and discriminating power indices. Micro features were examined using the four most frequently appearing letters: “a”, “y”, “e” and “n”. A comparative analysis of tested Latin letters with those of a similar configuration in Cyrillic demonstrated differences in the number of distinguishing features, as well as in the frequency of occurrence and discriminating power indices of similar features. These results show that separate statistical bases should be used for Latin and Cyrillic letters when analysing handwriting samples based on the frequencies of occurrence of micro and macro writing features.

## Key points

The Republic of Kazakhstan is currently moving from the use of a Cyrillic alphabet to a Latin one.

Kazakhstani document examiners are faced with new difficulties in the identification of handwriting samples based on Latin script.

In this article, 100 Kazakh-language handwriting samples prepared using Latin letters were obtained from Kazakh nationals and analysed.

Distinguishing macro and micro features within these samples were identified and their frequencies of occurrence and discriminating power indices were calculated.

Results from this study show that features of Latin letters should be analysed using a separate statistical base from that used for Cyrillic.

## Introduction

Since 2017, the Kazakh alphabet has been in the process of being transferred from Cyrillic to Latin [1]. Consequently, questioned document examiners in the Republic of Kazakhstan will soon be faced with examining new types of documents in Latin script. However, an analysis of existing methodologies in Kazakhstan shows an absence of any standardized protocol for the examination of handwriting samples produced in the Kazakh language using the Latin alphabet [2].

A prevailing hypothesis among experts from Russia and Kazakhstan is that handwritten manuscripts in Latin script, regardless of language, can be examined using the same approaches designed for the analysis of Cyrillic text. However, experiments conducted within the Kazakhstan Ministry of Justice have already shown that whilst letters in both scripts share some common macro and micro features, challenges arise in the analysis of Latin letters that do not possess a graphic analogue in Cyrillic (unpublished data). Furthermore, examiners may also face difficulties from a lack of knowledge in relation to the structure of written characters and the frequencies of occurrence and discriminating power indices of different features. Such difficulties may have a negative impact on the accuracy of an examiner’s conclusion and make any statements they produce unreliable. Moreover, experiments conducted in this area so far have only relied on qualitatively descriptive methods, without any calculation of the discriminating power of studied features (a tool that may potentially be used to provide a quantitative basis for examination).

Recently, the reliability of handwriting evidence has been called into question by courts [3, 4], making the objectification of this type of analysis extremely important. A series of studies have therefore been undertaken in recent years with the express purpose of building a statistical base of the frequencies of occurrence of different handwriting features, which may be used by questioned document examiners to establish the authorship of a manuscript through the identification of particularly discriminatory macro and micro features. In countries with the Latin alphabet, studies have already been conducted on the structural features of certain letters, including the letters “a”, “d”, “y”, “f”, and the grapheme “th” [5]. The frequencies of occurrence of selected features in all letters of the English language within handwriting samples obtained from US citizens have also been measured [6, 7].

Whilst experts within the Republic of Kazakhstan have been using methods for establishing manuscript authorship based on frequencies of occurrence and discriminating power indices of Cyrillic letters since the 1960s [8], there are currently no similar guidelines for the analysis of documents written in the Kazakh language using the Latin alphabet. Therefore, the aim of this study is to provide a statistical base of frequencies of occurrence and discriminating power indices of macro and micro handwriting features of Kazakh-Latin letters through the analysis of 100 medium-text handwriting samples obtained from Kazakh nationals.

## Materials and methods

### Character selection

For this work, only the most commonly occurring letters present in the Kazakh language written in Latin script were investigated (due to the fact that they are likely to appear not only in large and medium volumes texts, but also in short notes). To determine the frequency of occurrence of each letter in the alphabet, 30 medium- and large-sized newspaper articles (more than 500 written characters) made in Kazakh using Latin letters were analysed. The four most frequently appearing characters were determined as “a”, “y”, “e”, “n” (Table 1).

**Table 1.** The relative frequencies of occurrence of the characters of the Kazakh language written in Latin script from analysed texts.

Character	Frequency occurrence in texts (%)
a	100.0
y	90.0
e	86.6
n	73.3

Further study of macro and micro features was conducted for each of the selected letters regardless of their location within a word (i.e. at the beginning, middle or end of the word).

### Sample collection

A total of 100 handwriting samples were collected from male and female individuals, aged between 22 and 59 years, of Kazakh nationality. Whilst all participants were current staff members of the Kazakhstan Ministry of Justice and therefore possessed a working knowledge of forensic science, none of the individuals were formally trained in forensic handwriting analysis methods. No other participant selection conditions, including dominant writing hand, educational background or literacy level, were imposed.

Participants were asked to copy a set portion of text (more than 500 written characters) selected from a newspaper article that contained multiple instances of all previously selected frequently occurring characters. Handwriting samples were obtained under ordinary conditions, with all participants sat in a usual writing position, at a table and under natural or artificial light. Text was written with conventional writing instruments, mainly ballpoint pens, on specially prepared white standard non-lined sheets of medium-density paper. The time to complete the manuscript was not limited.

### Feature extraction

Macro and micro feature analysis was carried out separately by the same experienced questioned document examiner on obtained handwriting samples. All examinations were performed manually (i.e. by eye and without the use of computer software). All texts were analysed in a randomized order.

For macro features of handwriting, a table of experimental results was compiled, consisting of four columns:

- Name and structure of macro feature
- The sample number(s) in which the feature occurs
- The total number of samples in which the feature occurs
- The percentage of occurrence of the feature within studied samples

All possible variants of macro features of handwriting [9–14] were listed in the 1st column of the table. Then, in the process of studying each obtained handwriting sample, the number of the sample in which specific variants of macro features appeared was added to the 2nd column. Calculations were then made to establish the total number of samples in which a specific featured occurred and the percentage occurrence of the feature within all studied samples (which were entered the 3rd and 4th columns of the table, respectively).

For micro features, the processing of handwriting samples was carried out as follows: for each character, eight tables were developed (one for each “group” of micro handwriting features studied [15]), each consisting of five columns:

- Description and graphic sketch of the feature
- The sample number(s) in which the feature was found to occur
- The total number of samples in which the feature occurred
- The percentage of occurrence of the feature within studied samples
- The discriminating power index of the feature

In order to be classified as a true occurrence, micro features within a given sample of text must have been able to satisfy the following conditions:

1. Be stable, repeating in the text at least 3–4 times.
2. Not be indicative of the manuscript being written in unusual conditions or using intentionally disguised handwriting.
3. Be accurate as described and not an approximate version of the feature.
4. Be consistent with the description of the feature, and not contain any additional elements outside of this description.

Two summary tables containing a list and detailed description of all identified macro and micro features can be found in the associated Supplementary Materials for this article.

### Data analysis

The absolute and relative frequencies of occurrence of macro and micro features, respectively, as well as the discriminating power of micro features was calculated and compiled into two summary tables (Supplementary Materials). The calculation of absolute frequencies of occurrence of features was carried out by simple arithmetic summing up all appearances of the feature in studied manuscripts. These proportions were entered in the appropriate columns in the tables for macro and micro features. Relative values of the frequencies of occurrence of macro and micro features were determined by using the formula:

W=m/n

where “*W*” is the relative frequency of occurrence of a feature; “*m*” is the number of samples in which the feature appeared, and “*n*” is the total number of experimental samples. Based on obtained frequency of occurrence values, calculations of the discriminating power indices of micro features (also known as “negative log probability”) were carried out according to the following formula [16]:

L = −lg P

Here, the “discriminating power index” of a micro feature (“*L*”) is defined as the negative decimal logarithm (“−*lg*”) of the probability of occurrence (relative frequency of occurrence) of the feature (*P*). The frequency of occurrence (absolute or relative) and the discriminating power index of a feature are therefore inversely proportional. The more often a feature occurs, the less its discriminating power and conversely, the less it occurs in the analysed group of handwritings, the more valuable it is for identification (and the higher its discrimination index value). Such indices may consequently be used by questioned document examiners to determine the relative “rarity” of a writing feature within a specific population and provide a quantitative basis for author identification.

## Results

Experiments were conducted to determine the frequency of occurrence and discriminating power indices of the features in the four most common letters of the Kazakh language written using the Latin alphabet. During this study, only micro traits deviating from writing norms were considered.

### Macro features

Macro features were considered in three groups (each of which has separate subgroups): features that reflect the quality of writing, line quality and motor skills; features that characterize the structure of movements on their trajectories and features that characterize the spatial orientation of movements (see Supplementary Materials for a full list of examined macro features and associated descriptions).

From the analysis of obtained handwriting samples, it was determined that the most common macro features related to writing quality, line quality and motor skills were: a higher average degree of quality of writing (59%), an average speed of writing (40%), a higher average line quality of movement (50%) and a simple structure (88%). Conversely, the least common macro features observed within this group was handwriting characterized by an average degree of quality of writing (14%), below average speed of writing (1%), below average line quality (2%) and complicated structure (1%).

Within analysed samples, the most common macro features characterizing the structure of movements on their trajectories were: the predominant shape of strokes — “arcuate in different configurations” (55%), the predominant direction of strokes — “counter clockwise” (73%), slant — “right in different configurations” (66%), size —“within average” (64%), acceleration — “average” (84%), arrangement of written signs — “narrow” (73%), degree of connectivity — “from interval to small” (32%), pressure “average” (74%) and “differentiated” (94%). Also in this group, the following features were characterized as the least frequently occurring: the predominant shape of strokes — “mixed with “printed” handwriting” (1%) and “sinuous-squared” (1%), the predominant direction of movement — “left-circular with the presence of right-circumferential elements” (27%), slope — “vertical” (5%), size — “unstable large” (2%), acceleration — “large” (3%), arrangement of written signs — “unstable” (27%), pressure — “weak” (5%) and “undifferentiated” (6%).

It was also discovered that the most frequently occurring macro features characterizing the spatial orientation of movements were: the presence of only left margins (64%), presence of paragraphs (85%), average size of intervals between rows (42%), average size of intervals between words (79%), straight line shape of the letter line (77%), horizontal direction of the letter line (39%), placement of punctuation marks (on the line of the letter (47%), at the average distance relative to the preceding word (70%), in the middle of the gap relative to the centre between the preceding and subsequent word (51%) and placement of hyphenation characters (on the middle line of the letter (26%), with a horizontal direction (40%). Within this group of macro features, the lowest frequently occurring were found to be: the presence of left and right margins (5%), no paragraph highlighting (15%), unstable line spacing — “medium to large” (4%), unstable size of intervals between words—from small to medium (3%), the sinuous shape of the letter line (1%), the direction of the letter line—from horizontal to descending (3%), placement of punctuation marks (the line of letters at the same time on the line below and above (2%), relative to the preceding words — “at close distance” (1%), the large and very large distance (1%), relative to the centre between the preceding and the following word — “close word” (4%) and placement of hyphenation characters (above and below the middle line of the letter (4%), the direction of which is rising and falling (1%)).

### Micro features

Micro features were considered for the four most common letters “a”, “y”, “e”, “n”. Except for “e”, each of these letters consist of two structural elements. Through the analysis of obtained handwriting samples, it was determined that some micro feature groups, such as “sequence of movements at performance”, were not observed in any of the studied letters. In addition, the letter “e” was not found to contain any variable micro features from the group “complexity of movements” (see Supplementary Materials for a full list of examined micro features and associated descriptions).

**Figure 1.**
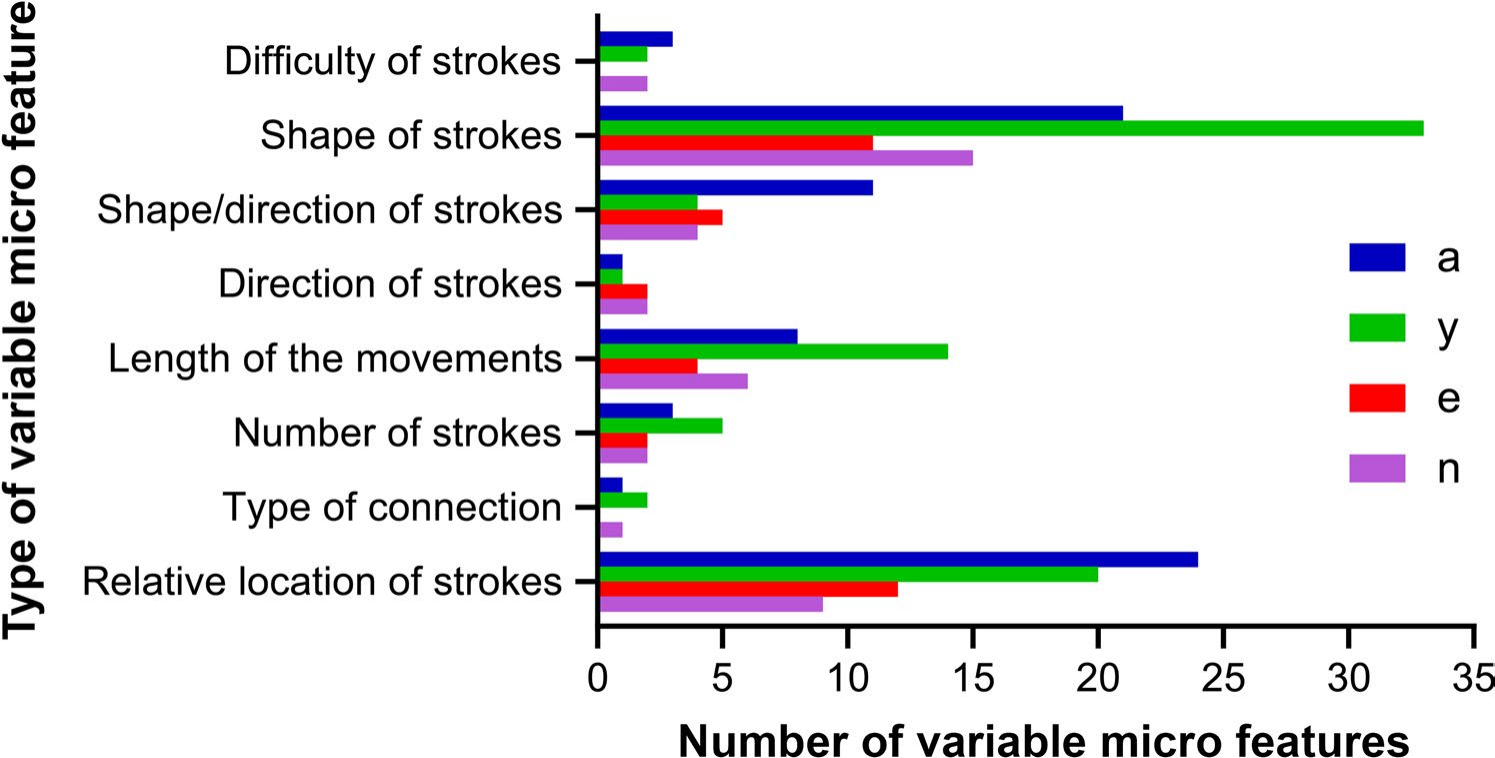
The number of variants identified within each micro feature group for all four studied letters from obtained Kazakh language handwriting samples.

For each letter, a different number of micro features were identified, as demonstrated in Figure 1, the letter “y” showed the largest number of total variable micro features (*n* = 81), while in the letter “e”, only 36 variable features were detected.

As demonstrated in Table 2, 19 out of the 72 variable micro features identified for the letter “a” were shown to occur at low frequencies and may therefore be considered to possess high discrimination power, with *L* values between 1.70 and 2.00. Conversely, five features with high frequency of occurrence and low discriminating power (*L* values between 0.08 and 0.28) were identified.

**Table 2.** The number and percentage of micro features of the letters “a”, “y”, “e” and “n” identified within studied Kazakh language ­handwriting samples grouped by discriminating power index (*L* value).

a (*N* = 72)		y (*N* = 81)		e (*N* = 36)		n (*N* = 41)	
*L* value	*n* (%)^a^	*L* value	*n* (%)^a^	*L* value	*n* (%)^a^	*L* value	*n* (%)^a^
2.00	7 (9.7)	2.00	8 (9.9)	2.00	1 (2.8)	2.00	1 (2.4)
1.70	12 (16.7)	1.70	1 (1.2)	1.70	3 (8.3)	1.40	2 (4.9)
1.52	3 (4.2)	1.52	7 (8.6)	1.52	2 (5.6)	1.22	3 (7.3)
1.40	3 (4.2)	1.50	3 (3.7)	1.40	3 (8.3)	1.15	2 (4.9)
1.30	5 (6.9)	1.30	8 (9.9)	1.30	1 (2.8)	1.10	1 (2.4)
1.22	2 (2.8)	1.22	2 (2.5)	1.05	1 (2.8)	1.05	1 (2.4)
1.15	4 (5.6)	1.15	2 (2.5)	0.92	1 (2.8)	0.92	1 (2.4)
1.10	3 (4.2)	1.10	4.9)	0.89	1 (2.8)	0.89	1 (2.4)
1.05	1 (1.4)	1.05	2 (2.5)	0.85	1 (2.8)	0.85	1 (2.4)
1.00	1 (1.4)	1.00	2 (2.5)	0.82	1 (2.8)	0.82	1 (2.4)
0.96	3 (4.2)	0.96	3 (3.7)	0.77	1 (2.8)	0.74	1 (2.4)
0.89	2 (2.8)	0.92	1 (1.2)	0.70	1 (2.8)	0.72	1 (2.4)
0.82	1 (1.4)	0.89	1 (1.2	0.62	1 (2.8)	0.68	1 (2.4)
0.80	1 (1.4)	0.85	2 (2.5)	0.57	2 (5.6)	0.66	2 (4.9)
0.77	2 (2.8)	0.82	1 (1.2)	0.51	1 (2.8)	0.64	1 (2.4)
0.70	1 (1.4)	0.80	4.9)	0.44	1 (2.8)	0.55	1 (2.4)
0.55	3 (4.2)	0.77	2 (2.5)	0.43	1 (2.8)	0.42–0.49	4 (9.8)
0.52	1 (1.4)	0.74	2 (2.5)	0.30–0.37	4 (11.1)	0.31–0.39	5 (12.2)
0.42–0.49	7 (9.7)	0.70	1 (1.2)	0.05–0.22	9 (25.0)	0.01–0.29	11 (26.8)
0.30–0.38	5 (6.9)	0.68	1 (1.2)	–	–	–	–
0.08–0.28	5 (6.9)	0.41–0.49	6 (7.4)	–	–	–	–
–	–	0.33–0.38	4 (4.9)	–	–	–	–
–	–	0.03–0.24	14 (17.3)	–	–	–	–

aPercentages might not total 100% due to rounding.

Despite the fact that the letter “y” ranked first among the studied letters in terms of the total number of micro feature variants identified, only nine traits displayed sufficient discriminating power indices, with *L* values between 1.70 and 2.00, whilst 14 traits demonstrated low indices, with *L* values between 0.03 and 0.24 (Table 2).

For the letter “e”, four micro features possessed high discriminating power indices, with *L* values from 1.70 and 2.00, whilst nine features frequently occurred and therefore possessed poor identification information content, with *L* values between 0.04 and 0.22 (Table 2).

Only three out of 41 micro features identified for the letter “n” were found to occur infrequently, possessing *L* values between 1.40 and 2.00. Whereas, only 11 traits with high frequency and low discriminating power values (*L* values between 0.01 and 0.29) were identified (Table 2).

After determining the discriminating power indices of all micro features for each of the four letters studied, the proportional ratios of high- and low-informative features were calculated (for example, in the letter “a”, approximately 26% (19/72) of the total number of identified micro features were found to have high discriminating power, whilst only 14% (10/72) of features showed low indices). The results collated as part of Tables 1–2 demonstrate a direct relationship between the frequency of occurrence of letters and their discriminating power index (i.e. the most frequently occurring letters have a larger number of micro features with highly informative indices).

## Discussion

As a result of the analysis of obtained Kazakh language handwriting samples, tables of the frequencies of occurrence of macro and micro features, as well as the discriminating power indices of micro features, have been compiled (Supplemental Materials). These tables may be used by questioned document examiners in the process of determining the authorship of a handwritten manuscript written in the Kazakh language using Latin script when using analysis methods based on the statistical frequency of occurrence of specific macro and micro features (although such methods are not yet employed by questioned document examiners in every country).

It is believed that lowercase letters in Latin script with upper and lower loops have more identification significance than those without [5]. Within this study, micro feature analysis was carried out in relation to the most common letters, three of which do not have any loops. Nevertheless, the results of this work are still likely to have practical benefits for handwriting examiners, as there is a high probability that these letters will appear in texts of small or medium length. As a result, it is pertinent for experts to be aware of the most informative features of these letters.

Mathematical methods of author identification, as a rule, are based on the detection of unique combinations of macro and micro features that are implicit to handwriting samples produced by a specific individual. According to the rules of probability theory, the likelihood of a handwriting sample belonging to another individual is reduced each time when a new micro or macro feature is identified within that sample. This pattern corresponds to the rule of multiplication of probabilities, i.e. the probability of the joint occurrence of several events is equal to the multiplication of the probabilities of each event [17]:

Р(АВ) = Р(А) х Р(В)

where P(A) is the probability of occurrence of event A and P(B) is probability of occurrence of event B. Therefore, in author identification, the frequency of the simultaneous occurrence of specific macro/micro features is equal to the multiplication of the frequencies of occurrence of individual features:

W(А В С … N) = W(А) х W(В) х W(С) х …… х W(N)

Through this formula, the probability of coincidence (frequency of occurrence) of sets of features can be determined. However, in handwriting analysis, experts are typically tasked with the identification of a large number of features, which makes the process of multiplying many smaller units cumbersome. Therefore, the logarithm of expression is used:

lg Р (А В С … N) = lg Р(А) + lg Р(В) + lg Р(С) +…… + lg Р(N)

Inputting discriminating power indices into this formula subsequently produces:

L(А В С…N) = L(А) + L(В) + L(С) +…….+ L (N)

where *L*(A), *L*(B), *L*(C),…, *L*(*N*) are the discriminating power indices of individual features and *L* (A B C…*N*) is the sum of the discriminating power indices of all features included in a sample. However, in practice, this formula may only be applied under the following basic conditions:

1. The writings under study must not be of a low quality (as judged by the examiner). Among the 100 samples analysed, none were considered as having a low degree of quality of writing.
2. The questioned writing must have been made under normal conditions. When the expert detects signs of unusual writing conditions or the abnormal state of an individual at the time of writing, this statistical method is not applicable.
3. A piece of writing should be written in the Kazakh language using Latin script.
4. An expert must empirically conclude that the writing sample is genuine. This method does not apply for forged subjects.

If a writing sample meets all the above conditions, an expert can proceed to the main stage of analysis in which individual letters are investigated. If a macro or micro feature is found to be well expressed, stable and accurately matches its given description, its discriminating power index value (as indicated in the compiled table) should be taken. However, this is also dependent on three specific factors:

1. Only one feature should be taken per analysed letter (i.e. the feature with the strongest discriminating power). This is done to prevent the co-identification of interdependent features, whose occurrence probabilities cannot be considered as separate events.
2. Micro features of handwriting defined should not be repeated within corresponding macro features. For example, if the predominant shape of movements in the writing are curved and looped, micro features based on arcuate or loop-shaped movement cannot be considered.
3. The independence of micro features from each other should be considered. Interdependence of micro features is defined as the presence of the same features in similar elements of different letters.

**Figure 2.**
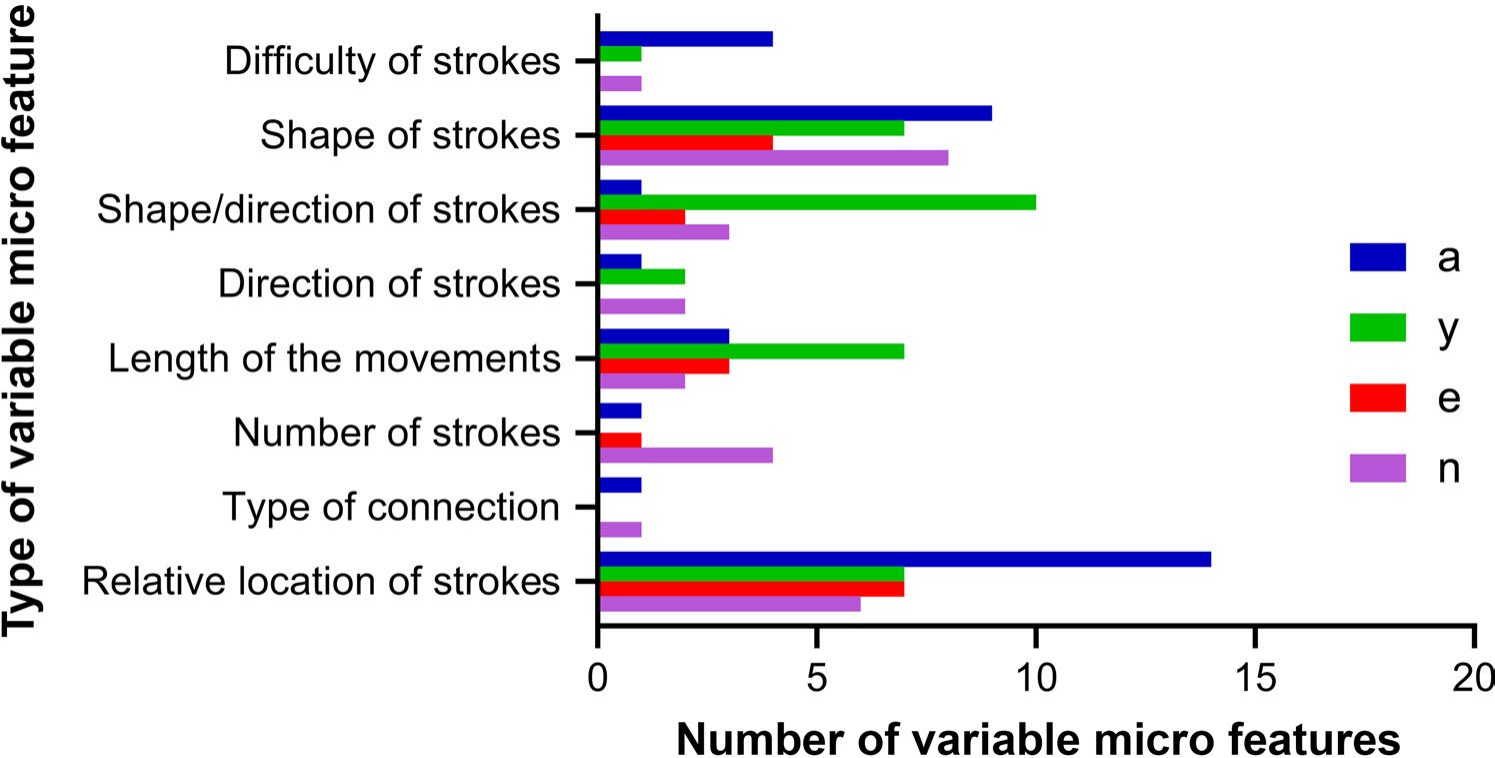
The number of variants identified within each micro feature group for all four studied letters in Cyrillic script.

Since the Latin letters “a”, “у”, “e” and “n” analysed during this study have a similar structure and configuration to their analogues within the cursive Cyrillic alphabet, it is possible to compare the discriminating power indices of similar micro features across both scripts. The number of variants within each micro feature group was found to be less in Cyrillic compared to Latin for all four letters (Figure 2).

Differences were also observed in the frequencies of occurrence and discriminating power indices of similar micro features across both alphabets. For example, one micro feature characterizing the complexity of movements performing the letter “a” (due to the repetition of the oval), was detected in both Cyrillic and Latin, but the discriminating power indices for this feature was found to be different for each script (0.74 and 0.48, respectively). In general, discriminating power indices for similar features in Latin were found to be lower than in Cyrillic. This may be explained by differences in the writing system and qualities of writing in Latin among the tested people compared to Cyrillic (since all tested people developed their writing skills based on Cyrillic and continue to use Cyrillic in everyday practice). These findings also lend additional support to the theory that handwriting analysis based on the frequencies of occurring character features may be used to discriminate between authors of different geographical populations [5]. Whilst author identification of Latin handwriting based on comparative analysis with similar letters in Cyrillic is possible using mathematical methodologies, it should be borne in mind that to reach a strong and reliable conclusion, discriminating power indices determined for the Latin alphabet should be used. Therefore, there is a need of further work to identify the frequencies of occurrence and discriminating power indices of micro features for other letters of the Kazakh language in the Latin alphabet, in order to create a basis for the application of the statistical method to author identification, which will be imminent for handwriting examination in Kazakhstan.

## Conclusions

A study of the macro and micro features of handwriting in the Kazakh language prepared using Latin script was conducted. Micro features were examined in the four most frequently appearing letters: “a”, “y”, “e” and “n”. Measurements of the relative frequency of occurrence of both types of features, as well as the discriminating power indices of micro features for the tested letters, were calculated. These calculations were based on the analysis of 100 handwriting samples in the form of large-volume text in the Kazakh language, written in Latin script by Kazakh nationals.

An analysis of identified features revealed the following insights:

1. A large number of micro features among tested letters belonged to the letter “y” (81). This may be explained by the structural differences between both cursive and printed (simplified) versions of the letter, which were both present within written samples.
2. Specific micro feature groups, such as “relative location and the shape of movements”, contained a greater number of variants than others.
3. A direct relationship was found between the frequency of occurrence of letters and the discriminating power of their features (i.e. the most frequently appearing letters possessed larger proportions of highly informative features).

Furthermore, the features and conditions for applying statistical methods towards the analysis of Latin script handwriting samples were determined. In particular, questioned pieces of writing should be written in Kazakh language by a person whose degree of quality of writings should not be low and not contain signs of unusual writing performance. Empirically, an expert must also conclude that a writing sample is genuine, as well as take into account the interdependence of different macro and micro features.

A comparison of tested Latin letters with their Cyrillic analogues showed differences in the total number of identified features, as well as in the frequency of occurrence and discriminating power indeces of similar features. A greater number of micro feature variants were seen in Latin letters. However, discrimination values were generally lower than those of Cyrillic counterparts. This may allow experts to establish authorship of Latin texts using approaches already established for Cyrillic. However, in order to make a conclusion more accurate and reliable, an expert should use discrimination values corresponding to Latin letters. Therefore, further work is necessary to identify the frequency of occurrence and the discriminating power of other Latin letters of the Kazakh language.

## Supplementary Material

Supplemental Material
